# Awareness, use and associated factors of emergency contraceptive pills among women of reproductive age (15-49 years) in Tamale, Ghana

**DOI:** 10.1186/1472-6874-14-114

**Published:** 2014-09-22

**Authors:** Anthony Amalba, Victor Mogre, Monica NA Appiah, Winnifred A Mumuni

**Affiliations:** Department of Human Biology, School of Medicine and Health Sciences, University for Development Studies, P. O. Box TL 1883, Tamale, Ghana; Department of Allied Health Sciences, School of Medicine and Health Sciences, University for Development Studies, Tamale, Ghana

**Keywords:** Emergency contraceptive pills (ECPs), Availability, Affordability, Cultural, Religious, Women of reproductive age, Tamale, Ghana

## Abstract

**Background:**

Emergency contraceptive pills (ECPs) are one of the means by which women can use after intercourse to prevent pregnancy. ECPs can be used to reduce the prevalence of unwanted pregnancies and unsafe abortions. This study investigated awareness and use of ECPs among reproductive age (15-49 years) women in Tamale, Ghana. Factors associated with the use of ECPs were also investigated.

**Methods:**

This cross sectional study was conducted among 200 women of reproductive age (15-49 years) in Tamale, Ghana. Data on socio-demographic variables, awareness and usage of ECPs were assessed by means of a previously validated questionnaire. Univariate and multivariate logistic regression analyses were performed to identify factors associated with the use of ECPs.

**Results:**

Awareness level of ECPs were found to be 69.0% (n = 138); 42.8% (n = 59) got the awareness from a health worker, 31.8% (n = 44) from the radio/TV and 25.4% (n = 35) from family members/friends. Eighty-five percent (n = 117) knew the correct time-frame for an effective use of ECP to prevent pregnancy. Forty percent (39.9%, n = 55) of the participants who had awareness have ever used ECPs. Factors that were found to be associated with the use of ECPs were; participants who said ECPs were affordable (AOR = 6.1, 95% CI = 2.51 – 10.40, p = 0.001), available (AOR 2.1, 95% CI = 0.61 – 6.01, p = 0.001), cultural (AOR = 3.5, 95% CI = 1.01 – 10.15, p = 0.011) and religious unacceptable (AOR = 4.0, 95% CI = 1.02 – 10.0, p = 0.005).

**Conclusion:**

A relatively high level of awareness and usage of ECPs was found. Factors that were associated with the use of ECPs were availability and affordability. Cultural and religious unacceptability did not hinder the use of ECPs. Health authorities should continue to make ECPs available to women of reproductive age.

## Background

Emergency contraception (EC) is defined as any method women can use after intercourse to prevent pregnancy [1]. The differing methods of EC include the use of intrauterine devices and hormone pills (referred to as emergency contraceptive pills (ECPs), such as the over-the-counter hormonal method known as Plan B [2]. The most commonly used EC method in developing countries including Ghana is the ECPs.

ECPs referred to as postcoital contraception are mostly hormone-based regimens, including a combination of ethinyl estradiol with levonorgestrel (Yuzpe regimen) and levonorgestrel alone [3], Ulipristal acetate30 mg [4]. Just like any other EC method they are administered after unprotected intercourse. In the past, ECPs were considered to be effective only within 72 hours after intercourse; however recent studies have indicated that they are effective for up to 120 hours [5–7]. Their effectiveness has been claimed to be between 75–80 percent [8].

Increasing the availability and promotion of EC has the potential to reduce the incidence of unintended pregnancies [9–11]. Uniquely, it is the only immediate option left for a woman who has had unprotected intercourse and is unprepared for a pregnancy [12].

Each year, about 210 million women around the world become pregnant [6] from which about 75 million of these pregnancies (36%) are unplanned and/or unwanted [13]. Unplanned/unwanted pregnancy is one of the leading causes of maternal mortality and morbidity in developing countries [6]. In the developing countries, the World Health Organization (WHO) estimates that one woman dies every eight minutes due to unsafe abortion [14, 15].

In Ghana, ECPs have now become an integral part of contraceptive services to prevent conception following unprotected and unplanned exposure or contraceptive accidents like burst condom, slippage of diaphragm and forgotten pills. Also ECPs are used by victims of rape cases. There has been very extensive campaign and dissemination of knowledge on contraceptives. Currently, the knowledge of any contraceptive method is almost universal in Ghana, with 98 percent of all women and 99 percent of all men knowing at least one method of contraception [16]. However, while 47.4% of Ghanaian women have a history of contraceptive use only 20% are current users [16].

Since the introduction of ECPs in Ghana, limited studies have been conducted to assess awareness and usage of ECPs among women of reproductive age. The few studies available were conducted among college students.

This study investigated the awareness and use of ECPs among reproductive age (15–49) women in Tamale, Ghana. Furthermore factors associated with the use of ECPs were investigated.

## Methods

### Participants

This cross-sectional study was conducted from April to July, 2012 among reproductive age women (15-49 years) in Tamale, Ghana. Tamale is the capital city of the Northern region of Ghana, located about 500-600 km North of Accra, the Capital city of Ghana.

During the study, Tamale was clustered into three electoral constituencies (Tamale central, north and south) from which 200 participants were included in the study. Purposively, 115 women were approached in Tamale central, 100 consented to the study yielding a response rate of 87.0%. From Tamale North and South, 53 and 55 women respectively were approached, from which 50 women each from North and south consented to the study (response rate of 94.3% and 90.0% respectively). The inclusion of participants was voluntary and informed consent was obtained from each participant. Participants were also assured of confidentiality of any data taken. All participants were approached in their homes. The study was approved by the Ethics Committee of the University for Development Studies, School of Medicine and Health Sciences, Ghana.

### Questionnaire

A previously validated [6, 17], 10-item questionnaire was developed to collect information on participants’ knowledge and usage of ECPs as well as associated factors. Data on socio-demographic variables such as gender, age and educational status were obtained. Cultural and religious acceptability as well as availability and affordability were assessed using yes/no questions that were included in the questionnaire. For example, “does your religion accept the use of ECPs? Yes or No?” Awareness was assessed by the question; “have you ever heard of EC? Yes or No”. Knowledge levels of participants were assessed using questions relating to the appropriate time to use ECPs. Format of the knowledge questions included single statements yes or no questions and multiple choice questions. Participants were asked to choose the best answer in response to the multiple choice questions. All questionnaires were anonymous and did not have any identifiers. In order to ensure confidentiality all the questionnaires were administered to the participants by a female research assistant in a well secluded area, free from any form of disruption.

### Statistical analysis

All statistical analysis was conducted using GraphPad Prism version 5 (GraphPad software, San Diego California USA, http://www.graphpad.com/scientific-software/prism/) and two-tailed *p* values. The results were expressed as proportion and compared using Fisher’s exact test or χ^2^ for trend analysis as appropriate. Univariate logistic regression analysis was conducted to identify factors associated to the use of ECPs. Significant independent variables from the univariate analysis were included into a multivariate model and analysed. A level of p < 0.05 was considered as statistically significant. The educational status of the participants was classified into either “low” or “high” educational status. Participants who had primary and junior high level of education were combined to form “low” educational status. Those who had attained senior high level of education and beyond were considered as “high” level of education.

## Results

The general characteristics of the women are presented in Table 1. Generally, 67.5% (n = 135) had attained a high level of education, 84.5% (n = 169) were married, 44.0% (n = 88) self-employed, 49% (n = 98) Muslims and 34.5% (n = 69) had an income level below 200GHC ($ 77). The cost of ECPs ranged from 5GHC to 20GHC ($1.5 to $6) depending on the brand.

**Table 1:** **Socio-demographic characteristics of the women**

Variable	Frequency	%
**Age (years)**		
15-29	101	50.5
30-49	99	49.5
**Educational status**		
Low	65	32.5%
High	135	67.5%
**Marital status**		
Married	169	84.5
Not married	31	15.5
**Occupational status**		
Unemployed	53	26.5
Self-employed	88	44.0
Civil servant	59	29.5
**Religious status**		
Christianity	95	47.5
Moslem	98	49.0
Traditionalist	7	3.5
**Monthly income**		
Below GHC 200	69	34.5
Between GHC 200-500	50	25.0
Above GHC 500	57	28.5
None	24	12.0

### Awareness, knowledge and usage of emergency contraceptive pills (ECP)

Presented in Table 2 are women’s’ awareness, knowledge and usage of emergency contraceptive pills (ECPs). One hundred and thirty-eight women (69.0%) had awareness on ECPs, from which 42.8% (n = 59) said they got the awareness from a health worker, 31.8% (n = 44) from the radio/TV and 25.4% (n = 35) from family members/friends. About 85% (n = 117) of those who had awareness on ECPs, knew the correct time-frame for an effective use of ECPs to prevent pregnancy recognizing the need to take the first dose within 72 hours after having unprotected sexual intercourse. As presented in Table 2, 50.0% (n = 69) of those aware of ECPs said they would use ECP in case of breakage of a condom during sexual intercourse;17.4% and 21.9% (78.8%) chose the options that that ECPs can be used in case of rape and failure to follow regular methods respectively.

**Table 2:** **Women’s awareness and knowledge levels and factors affecting usage of ECPs**

Variable	Frequency	%
**Awareness on ECPs**		
Yes	138	69.0
No	62	31.0
**Asked only among those reporting awareness (n = 138)**		
**Source of information**		
Health worker	59	42.8
Radio/TV	44	31.9
Family members/friends	35	25.4
*Time frame for use of ECPs		
Used 72 hours before sex	12	8.7
Used 72 hours after unprotected sex	117	84.7
Taken when pregnant	5	3.6
Taken everyday	4	2.9
*When would you use ECPs?		
After rape	24	17.4
When condom breaks	69	50.0
Forgot to take the pill	30	21.7
Undue pressure from partner	15	10.9
**Availability of ECPs**		
Yes	121	87.7
**Affordability of ECPs**		
Yes	127	92.0
**Cultural acceptance of ECPs**		
Yes	51	37.0
**Religious acceptance of ECPs**		
Yes	62	44.9
**Personal usage of ECPs**		
Ever used	55	39.9
**Asked only among those reporting use of ECPs (n = 55)**		
**Why did you use ECPs?**		
To prevent unsafe abortion	8	14.5
To prevent unwanted pregnancy	44	80.0
For birth spacing	3	5.5

*Women were asked to choose one answer or the best answer in response to this question.

Less than 13% (n = 17) said ECPs were not readily available to them; however 92.0% said it was affordable. With regards to cultural and religious acceptance, 63% (n = 87) said ECPs were not culturally acceptable, however 44.9% (n = 62) said the usage of ECPs was acceptable in their religion.

With regards to the usage of ECPs, 39.9% (n = 55) of the women had ever used ECPs, and they used it to prevent unsafe abortion (14.5%, n = 8), unwanted pregnancy (80%, n = 44) and for child spacing (5.5%, n = 3).

### Factors associated with the use of ECPs

Univariate logistic analysis was conducted to identify factors associated with the use of ECPs and presented in Table 3. Women who reported that ECPs were available had 4.8 times the odds of using them as women who reported they were not available. Other factors that were found to be associated with the use of ECPs were women who said ECPs were affordable (Crude OR = 7.0, 95% CI = 2.96 – 16.40, p = 0.001), culturally (Crude OR = 4.6, 95% CI = 1.63 – 12.78, p = 0.003) and religious unacceptable (Crude OR = 5.1, 95% CI = 1.97 – 13.33, p = 0.004).

**Table 3:** **Univariate analysis of factors affecting the usage of EC among the women**

Variable	n/N*	Rate of usage	Crude OR (95% CI)	P value
**Marital status**				
Married	45/116	38.8%	0.8 (0.30 – 1.91)	0.637
Not married	10/22	45.5%	1	
**Availability of ECPs**				
Yes	46/89	51.7%	4.8 (2.06 – 10.95)	0.001
No	9/49	18.4%	1	
**Affordability of ECPs**				
Yes	43/91	47.3%	7.0 (2.96 – 16.40)	<0.001
No	12/47	25.5%	1	
**Cultural acceptance of ECPs**				
Yes	5/31	16.1%		
No	50/107	46.7%	4.6 (1.63 – 12.78)	0.003
**Religious acceptance of ECPs**				
Yes	6/38	15.8%	1	
No	49/100	49.0%	5.1 (1.97 – 13.33)	0.004
**Age (years)**				
15-29	31/70	44.3%	1.5 (0.73 – 2.89)	0.301
30-49	24/68	35.3%	1	

*Number of participants with usage/number of subjects in each category.

Presented in Table 4 is the results of multivariate analysis of factors affecting the use ECPs, adjusting for age, marital status and socio-economic status. Availability of ECPs, its affordability as well cultural and religious unacceptability still remained significant predictors of the use of ECPs.

**Table 4:** **Multivariate analysis of factors affecting the use of ECPs among the women**

Variable	AOR (95% CI)	P value
Intercept		0.008
ECPs are available	2.1 (0.61 – 6.01)	0.001
ECPs are affordable	6.1 (2.51 – 10.40)	0.001
Cultural unacceptability	3.5 (1.01 – 10.15)	0.011
Religious unacceptability	4.0 (1.02 – 10.05)	0.005

Cox and Snell = 0.13, Nagelkerke = 0.23.

## Discussion

This study assessed the awareness, knowledge and use of emergency contraceptive pills (ECPs) as well as factors associated with its use among reproductive age women in Tamale, Ghana.

The awareness level of ECPs among the reproductive women in this study was found to be 69.0%, which is higher than the level reported among Egyptian women (24.5%) aged 19-49 years [14], among women in California (38.2%) aged 18-44 years [18] and 11.2% among educated women in India [19] and among university students in Ghana (43%) [20] and Cameroon (63%) [21]. Our findings however are lower than those reported among English reproductive women (90%) [22–24] and University students in the USA (94%) [25] and Jamaica (84%) [26].

In contrast to several studies [7, 14, 19, 27, 28], health workers were the major source of information on ECP for the study participants rather than radio/television and other sources like family and friends. However, our findings are in agreement with the results of Irfan and colleagues who found a large proportion of women saying they knew about EC through a health care provider [29].

Another important finding of this study was that, about 85% of the participants who had awareness on ECPs were able to determine the correct time frame for using ECPs. This is higher than the 38% reported among reproductive age women in California [18], 14.9% reported among female college students in India and 18% in University students in Nigeria [30]. Several studies have reported that many women who are aware of the existence of ECPs do not have adequate knowledge on the appropriate interval between unprotected intercourse and taking the ECPs [31, 32]. Probably, our findings could be attributed to the fact that a large proportion of the study participants had a high level of education. Also the participants indicated health workers as their major source of information on ECPs, which might have contributed to the high level of knowledge on the correct time frame to use ECPs. These factors have been shown to influence knowledge and awareness levels of ECPs in several studies [18, 19, 31].

In agreement with several findings we found that participants said they would use ECPs in cases of rape, unprotected intercourse, and failed regular methods [6, 7, 14]. This finding was also confirmed by the reasons participants gave for using ECPs which included prevention of unwanted pregnancies and unsafe abortions.

About 40% of the women who had awareness on ECPs had ever used them. This is higher than the 24.5% reported among Egyptian women aged 18-49 years [14]. The higher prevalence of ECP use in this study as compared to others studies [18, 19, 31] could be due to the fact that a large proportion of the women said ECPs were available (92.0%) and affordable (87.7%). Further confirming our findings, our univariate and multivariate logistic regression analysis indicated that participants who said ECPs were available and affordable were more likely to use them as compared to those who said it was not. This suggests that availability and affordability may be significant determinants of the use of ECPs. To increase the current usage prevalence and to consequently realize the benefits of ECP in reducing unwanted pregnancies and abortions, the health authorities should continue to make ECPs available to reproductive age women.

Surprisingly, women who said the use of ECPs were not acceptable in their culture and their religion was significantly more likely to use ECPs compared to their counterparts who said their culture and religion accepted it.

Most of the women interviewed in this study were either Muslims or Christians. In both religions, sex is generally considered for procreation. In Catholicism all forms of abortion and emergency contraception are prohibited [33] except for measures normally taken to save a mother that result in the death of the foetus [34]. In Islam abortion of a viable foetus is considered a serious crime equivalent to that of murder [35]. However, the prevailing view in Islam is that emergency contraception and abortion are permissible in certain situations [36]. Depending on the Islamic school and length of gestation of the foetus, religious opinion varies from unconditional permissibility to unconditional prohibition [37]. The values that an individual woman holds may not be in keeping with the documented official teachings of her religion or the expected cultural norms reported by other members of the same culture [38]. Through education and interaction with health workers, women have realized the need to live a quality life and also take good care of their children. This might have contributed to the high use of ECPs despite reporting religious and culturally unacceptability.

The use of ECPs by women against religious and cultural unacceptability could also be due to the notion that using ECPs is less unacceptable in comparison with other methods of terminating unwanted pregnancies such as abortion, which could easily be stigmatized.

It is worth noting the limitations of this study. Due to the nature of the cross-sectional design, cause and effect could not be investigated. Also the study participants comprised of reproductive age women in Tamale, which is an urban town. Hence it’s difficult to generalize the findings of this study to women from the rural communities of Ghana. However, this study is explorative in nature to evaluate the awareness and use of EC among women and forms as a basis for future elaborative studies.

## Conclusion

A relatively high awareness level and use of ECPs has been found. Factors found to be associated with the use of ECPs were availability and affordability. Religious and cultural unacceptability did not hinder the use of ECPs. Health authorities should continue to make ECPs available to reproductive age women.

## References

[1] Contraception E (1995). Consensus statement on emergency contraception. Contraception.

[2] Pruitt SL, Mullen PD (2005). Contraception or abortion? Inaccurate descriptions of emergency contraception in newspaper articles, 1992–2002. Contraception.

[3] Glasier A (1997). Emergency postcoital contraception. N Engl J Med.

[4] Fine P, Mathé H, Ginde S, Cullins V, Morfesis J, Gainer E (2010). Ulipristal acetate taken 48-120 hours after intercourse for emergency contraception. Obstet Gynecol.

[5] Schwarz EB, Gerbert B, Gonzales R (2007). Need for emergency contraception in urgent care settings. Contraception.

[6] Adhikari R (2009). Factors affecting awareness of emergency contraception among college students in Kathmandu, Nepal. BMC Women’s Health.

[7] Sychareun V, Hansana V, Phengsavanh A, Phongsavan K (2013). Awareness and attitudes towards emergency contraceptive pills among young people in the entertainment places, Vientiane City, Lao PDR. BMC Women’s Health.

[8] Trussell J, Ellertson C, Stewart F, Glasier A, Ketting E, Armstrong E, Robinson E, Metcalf-Whittaker M, Rivera R, Tadiar F (1996). The effectiveness of the Yuzpe regimen of emergency contraception. Fam Plan Perspect.

[9] Myer L, Mlobeli R, Cooper D, Smit J, Morroni C (2007). Knowledge and use of emergency contraception among women in the Western Cape province of South Africa: a cross-sectional study. BMC Women’s Health.

[10] Reynolds T (2002). Switching from prescription to over the counter. Ann Intern Med.

[11] Trussell J, Calabretto H (2005). Cost savings from use of emergency contraceptive pills in Australia. Aust N Z J Obstet Gynaecol.

[12] Byamugisha JK, Mirembe FM, Faxelid E, Gemzell-Danielsson K (2007). Emergency contraception and fertility awareness among university students in Kampala, Uganda. Afr Health Sci.

[13] WHO (2004). Unsafe Abortion-Global And Regional Estimates Of The Incidence Of Unsafe Abortion And Associated Mortality.

[14] El-Sabaa HA, Ibrahim AF, Hassan WA (2013). Awareness and use of emergency contraception among women of childbearing age at the family health care centers in Alexandria, Egypt. J Taibah Univ Med Sci.

[15] WHO (2003). Unsafe Abortion. Global And Regional Estimates Of The Incidence Of Unsafe Abortion And Associated Mortality In 2003.

[16] GDHS (2008). Ghana Demographic and Health Survey. Preliminary Report. Book Ghana Demographic and Health Survey. Preliminary Report (Editor ed.^eds.).

[17] Campbell JW, Busby SC, Steyer TE (2008). Attitudes and beliefs about emergency contraception among patients at academic family medicine clinics. Ann Fam Med.

[18] Foster DG, Harper CC, Bley JJ, Mikanda JJ, Induni M, Saviano EC, Stewart FH (2004). Knowledge of emergency contraception among women aged 18 to 44 in California. Am J Obstet Gynecol.

[19] Takkar N, Goel P, Saha P, Dua D (2005). Contraceptive practices and awareness of emergency contraception in educated working women. Indian J Med Sci.

[20] Baiden F, Awini E, Clerk C (2002). Perception of university students in Ghana about emergency contraception. Contraception.

[21] Kongnyuy EJ, Ngassa P, Fomulu N, Wiysonge CS, Kouam L, Doh AS (2007). A survey of knowledge, attitudes and practice of emergency contraception among university students in Cameroon. BMC Emerg Med.

[22] Ellertson C, Shochet T, Blanchard K, Trussell J (2000). Emergency contraception: a review of the programmatic and social science literature. Contraception.

[23] Smith BH, Gurney EM, Aboulela L, Templeton A (1996). Emergency contraception: a survey of women’s knowledge and attitudes. BJOG.

[24] Crosier A (1996). Womens knowledge and awareness of emergency contraception. Br J Fam Plann.

[25] Vahratian A, Patel DA, Wolff K, Xu X (2008). College students’ perceptions of emergency contraception provision. J Women’s Health.

[26] Harper CC, Ellertson CE (1995). The emergency contraceptive pill: a survey of knowledge and attitudes among students at Princeton University. Am J Obstet Gynecol.

[27] Aksu H, Kucuk M, Karaoz B, Oğurlu N (2010). Knowledge and attitudes of health care providers working in primary health care units concerning emergency contraception. Gynecol Obstet Investig.

[28] Langer A, Harper C, Garcia-Barrios C, Schiavon R, Heimburger A, Elul B, Reynoso Delgado S, Ellertson C (1999). Emergency contraception in Mexico City: what do health care providers and potential users know and think about it?. Contraception.

[29] Irfan F, Karim SI, Hashmi S, Ali S, Ali SA (2009). Knowledge of emergency contraception among women of childbearing age at a teaching hospital of Karachi. J Pak Med Assoc.

[30] Aziken ME, Okonta PI, Ande A (2003). Knowledge and perception of emergency contraception among female Nigerian undergraduates. Int Fam Plan Perspect.

[31] Novikova N, Weisberg E, Fraser I (2009). Does readily available emergency contraception increase women’s awareness and use?. Eur J Contracept Reprod Healthcare.

[32] Fagan EB, Boussios HE, Moore R, Galvin SL (2006). Knowledge, attitudes, and use of emergency contraception among rural western North Carolina women. South Med J.

[33] LoPresti AF, Manning C, Zuckerman P (2005). Christianity. Sex and Religion Toronto.

[34] Maguire DC (2003). Sacred rights: the case for contraception and abortion in world religions.

[35] Pennachio D (2005). Caring for your Muslim patients. Stereotypes and misunderstandings affect the care of patients from the Middle East and other parts of the Eslamic World. Med Econ.

[36] Maguire DC (2001). Sacred Choices: The Right To Contraception And Abortion In Ten World Religions.

[37] Omran A-R: *Family Planning In The Legacy Of Islam.**(United Nations Population Fund).* London and New York: Routledge; 2012.

[38] Srikanthan A, Reid RL (2008). Religious and cultural influences on contraception. JOGC-Toronto.

[CR39] The pre-publication history for this paper can be accessed here:http://www.biomedcentral.com/1472-6874/14/114/prepub

